# Routine patient assessment and the use of patient-reported outcomes in specialized palliative care in Japan

**DOI:** 10.1186/s41687-023-00565-z

**Published:** 2023-03-09

**Authors:** Nao Ito, Yoko Ishii, Maho Aoyama, Hirofumi Abo, Akihiro Sakashita, Yuko Matsumura, Mitsunori Miyashita

**Affiliations:** 1 Department of Palliative Nursing, Health Sciences, Tohoku University Graduate School of Medicine, Miyagi, Japan; 2 Iwate Medical University School of Nursing, Iwate, Japan; 3 Jichi Medical University School of Nursing, Tochigi, Japan; 4 Department of Palliative Medicine, Rokko Hospital, Hyogo, Japan; 5 Department of Palliative Medicine, Kobe University School of Medicine, Hyogo, Japan; 6 Department of Nursing, Kyoto City Hospital, Kyoto, Japan

**Keywords:** Palliative care, Patient-reported outcome measures, Patient-reported outcomes

## Abstract

**Background:**

Discrepancies in symptom assessment between providers and patients are reported in cancer care, and the use of patient-reported outcome measures (PROMs) has been recommended for patients receiving palliative care. However, the status of the routine use of PROMs in palliative care in Japan is presently unclear. Therefore, this study aimed to clarify this complex question. To this end, we administered a questionnaire survey either online or via telephone interviews (questionnaire: sent to 427 designated cancer hospitals, 423 palliative care units [PCUs], and 197 home hospices; interviews: conducted at 13 designated cancer hospitals, nine PCUs, and two home hospices).

**Results:**

Questionnaires were returned from 458 institutions (44% response rate). We found that 35 palliative care teams (PCTs, 15%), 66 outpatient palliative care services (29%), 24 PCUs (11%) and one (5%) home hospice routinely used PROMs. The most frequently implemented instrument was the Comprehensive Care Needs Survey questionnaire. Moreover, 99 institutions (92%) that routinely used PROMs responded these instruments as useful in relieving patients’ symptoms; and moreover, the response rate in regard to usefulness in symptom management was higher than that of institutions that did not routinely use PROMs (p = 0.002); > 50% of the institutions that routinely used PROMs stated that use of these instruments was influenced by disease progression and patients’ cognitive function. Moreover, 24 institutions agreed to be interviewed, and interviews demonstrated the benefits of and the barriers to the implementation of PROMs. Effective methods used in the implementation of PROMs were introduced as efforts to reduce the burden placed on patients and to promote healthcare providers’ education in the use of PROMs.

**Conclusions:**

This survey quantified the status of the routine use of PROMs within specialized palliative care in Japan, revealed barriers to wider PROM use, and identified needed innovations. Only 108 institutions (24%) routinely used PROMs within specialized palliative care. Based on the results of the study, it is necessary to carefully consider the usefulness of PROs in clinical palliative care, perform careful selection of PROMs according to the patient's condition, and evaluate how specifically to introduce and operate PROMs.

**Supplementary Information:**

The online version contains supplementary material available at 10.1186/s41687-023-00565-z.

## Background

Patient-reported outcomes (PROs) are defined as reports of a patient’s health condition that come directly from the patient, without any interpretation of the patient’s response by a clinician or anyone else [1]. Discrepancies in symptom assessment between providers and patients have been reported in cancer care, and the use of PROs has been recommended for patients receiving palliative care and chemotherapy [2, 3]. Moreover, in cancer care, PROs have been shown to improve communication between patients and healthcare providers, identify patient symptoms, improve patient assessment and care, and increase patient satisfaction [4–9]. Furthermore, patients undergoing routine PRO evaluations during chemotherapy have an improved quality of life and prolonged survival [7] (Additional file 1).

In a previous randomized controlled trial (RCT) conducted in outpatient cancer patients, 60% of patients were eligible for a PRO evaluation according to study inclusion criteria and reported symptoms when using the administered questionnaire [10]. Further, a systematic review conducted by a British research group reported that many PROs are administered in an outpatient setting [8]. Moreover, in an international palliative care setting, the most frequently used instruments were reported to be the European Organization for Research and Treatment of Cancer Quality of Life Questionnaire-Core 30 (EORTC QLQ-C30), the Edmonton Symptom Assessment System (ESAS), and the Palliative care Outcome Scale (POS) [8]. However, in palliative care clinical practice, it is estimated that about 60% of patients are unable to complete the relevant questionnaires due to the state of their health and related factors [11].

In Japan, the main providers of specialized palliative care services are palliative care units (PCUs), palliative care teams (PCTs), palliative care outpatient services, and home hospices. Specialized palliative care services provide care for patients with complex needs [12]. In a review article evaluating the POS, it was reported that the Support Team Assessment Schedule (STAS) was more frequently used in Japan and China; this scale collects information on healthcare provider assessments rather than PROs [13]. This finding was considered to be due to a preference for a paternalistic medical culture across the healthcare system in Japan. However, this finding may not reflect the actual clinical situation. For example, five years have passed since this survey, and screening of patient distress (whether through PROs or other modalities) has since become obligatory in Japanese designated cancer hospitals [14]. In addition, one of the PROMs CCNSq was developed in Japan. The CCNSq was developed with the aim of standardizing an evaluation tool for the assessment of symptoms in cancer patients eligible for palliative care in Japan and to screen for distress and identify symptomology in patients undergoing chemotherapy. This tool is also used in the consideration of the introduction of palliative care at an early stage. Moreover, the CCNSq has been shown to be reliable and valid in previous studies [15, 16]. The questionnaire form for the CCNSq includes the following fields: the area of most concern, an NRS, a Distress and Impact Thermometer, a request for consultation with a specialist team. Moreover, the momentum for PROs has been building in Japan (as successors to the Japanese-language version of the STAS, the STAS-J), and the Japanese version of the patient-rated Integrated POS (IPOS) has been verified for reliability and validity [17].

In the present study, we aimed to investigate the routine use of PROs in specialized palliative care in Japan. Moreover, in situations in which PROs were not implemented, we evaluated the reasons for not evaluating PROs. We also aimed to clarify the strategies implemented by the institutions that did evaluate PROs in order to devise new ways to use PROs in a wider range of settings. This study is the first report among routine patient assessment and the use of PROs in SPC in Japan and exploratory research.

## Methods

This study administered questionnaires and conducted online or telephone interviews. The questionnaires were sent to all institutions providing specialized palliative care in Japan and that were willing to participate in this portion of the study. The questionnaire surveyed respondents (healthcare providers) about their routine use of PROMs and their opinions on PROs. The interviews were conducted with informants who were healthcare providers. The interview asked healthcare providers for specific examples in regard to the usefulness of PROs as well as the barriers to their use; the interview also queried healthcare providers about effective methods used for collecting PROs. Respondents were to be representatives reporting on the institutional reality, not on their personal experience. Although we did not apply for ethical approval for this study, we conducted the study in accordance with Japanese research guidelines, including anonymity and respect for the respondents’ right to free will to participate in the study. For this study, the return of the questionnaire was regarded as the provision of consent for participation in the survey, and consent for participation in the interviews was obtained by e-mail or telephone. Participation in the survey was voluntary. All the survey results were statistically processed and the names of the hospitals were not disclosed to ensure ethical consideration for the respondents.

### Definition of terms

We defined “routine assessments” as regular daily or weekly evaluations during the course of inpatient or outpatient palliative care. Conversely, we defined “screening” as a single assessment, such as a distress screening at hospital admission or the patient’s first palliative care visit.

The PCT is a multidisciplinary team consisting of physicians, nurses, pharmacists, and other professionals such as psychologists and social workers that work across different hospital department [18]. The palliative care outpatient clinic provides to outpatients by physicians and nurses with expertise in palliative care. The PCU is a specialized ward for cancer and AIDS patients with the aim of providing them with a comfortable end of life. The home hospice provides in-home palliative care by home physicians and nurses.

### Settings and participants

Specialized palliative care service institutions (SPC) providing specialized palliative care in Japan were included in the present evaluation. In Japan, SPCs include PCTs, PCUs, home hospices, and palliative care outpatient clinics. All designated cancer hospitals have a PCT and a palliative care outpatient clinic. Moreover, screening for palliative care is mandatory for all cancer inpatient and outpatient settings of designated cancer hospitals since 2015 [14].

PCUs are required to admit patients with malignant tumors in need of pain management. Although there is no clear definition/regulatory designation for home hospice, in this study, home hospice was defined as an enhanced function home support clinic with a track record of emergency house calls and end-of-life care. Eligible institutions were identified from an open access list of palliative care service by the Ministry of Health, Labour and Welfare. In the implementation of this survey, administrators at designated cancer hospitals asked physicians or nurses on the PCT to respond to the survey. The questionnaire was mailed to 427 designated cancer hospitals, 423 PCUs, and 197 home hospices.

Of the institutions that responded to the study questionnaire, 13 designated cancer hospitals, nine PCUs, and two home hospices responded that they were willing to cooperate in the interview on the questionnaire were interviewed (i.e., a representative healthcare provider was interviewed) after reconfirming their willingness to cooperate in the study.

### Survey

#### Questionnaire survey

We administered the survey between September and December 2019. The questionnaire included the following items: facility background, evaluation tools used at the institution, whether PROMs or non-PROM evaluation tools were used, whether evaluation tools were used routinely, whether evaluation tools were used for screening, patient and provider opinions on PROs, if the facility had discontinued the use of PROMs, and the reasons for discontinuing the use of PROMs if so. We sent one reminder only to the institutions that did not respond to our initial contact (i.e., mailing the study questionnaire and relevant information). The questionnaire was developed through discussion by researchers who were experts in palliative care based on previous studies.

#### Interview

In addition to the study questionnaire, we conducted semi-structured interviews administered by trained researchers online or by telephone according to an interview guide prepared by our research team. Interviews were conducted by NI, who had interview training and experience in qualitative research. The researcher explained the purpose of the study to the participating institutions. Interviews were conducted between February and April 2020. The interview was administered to gather more insight into specific examples illustrating the usefulness of PROs as well as barriers to the effective collection and use of PROs in palliative care clinical practice; it also helped to gain a better understanding of information on the improvement of processes relevant to the administration of PROMs at each facility.

The interviews included questions relevant to the healthcare providers’ opinions on the use of PROMs for patients receiving palliative care, what the providers felt had been helpful or good about using PROMs for patients receiving palliative care, the usefulness of PROMs in informing care, and improvements in processes for administering PROMs at the healthcare providers’ institution (e.g., staff training and operational methods). The interviews were recorded on an integrated circuit (IC) recorder with the institution’s consent. The coding was compared and two authors (NI, YI) discussed discrepancies to inform the final coding with supervision.

### Data analysis

#### Questionnaire data analysis

We calculated descriptive statistics for each variable evaluated in the questionnaire survey according to the question/category. We performed Fisher’s exact probability tests for the PROMs that were routinely implemented at each institution, the reasons for discontinuing PROMs, and healthcare providers’ thoughts opinions in regard to PROs. We conducted two-tailed tests with a significance level of 5%. Of the returned questionnaires, those that answered at least 80% of the items were included in the analysis. Missing data were excluded. All analyses were performed using JMP 15 data analysis software (SAS Institute, Cary, NC, USA).

#### Interview data analysis

We also conducted a thematic content analysis of the interview data using verbatim transcripts of the recorded interviews [19]. We coded information on the usefulness of PROMs, barriers to the use of PROMs, and effective methods used for evaluating PROs, grouping similar semantic content according to categories and subcategories. The first two authors iteratively reviewed the analyses until they reached a consensus; another co-author (palliative care researcher M.M.) reviewed their selected categories and subcategories and judged them to be reliable.

## Results

### Questionnaire survey

#### Institution characteristics

We sent the study questionnaire to 1,047 institutions providing SPC in Japan and received valid responses from 458 institutions (representing a 44% response rate), with the following distribution: 227 designated cancer hospitals (50%), 212 PCUs (46%), and 19 home hospices (4%).

#### Routine use of PROMs and non-PROMs

The routine use of PROMs and non-PROM evaluations is shown in Table 1. Overall, only 108 (24%) of the surveyed institutions used PROMs, including 35 PCTs (15%), 66 outpatient palliative care services (29%), 24 PCUs (11%), and one home hospice (5%). Additionally, 93 PCUs (44%) routinely used non-PROM evaluation instruments such as STAS-J. In regard to screening, 76 PCUs (36%) used non-PROM instruments more often than PROMs for both routine assessments and when screening for patient distress.

**Table 1:** Routine use of PROMs and non-PROM PRO assessment methodologies

		Designated cancer hospital (n = 227)				PCUs (n = 212)		Home hospices (n = 19)	
		PCTs		Outpatient palliative care					
		n	%	n	%	n	%	n	%
Routine	PROMs	35	15	66	29	24	11	1	5
	Non-PROMs	51	22	40	18	93	44	1	5
Screening	PROMs	–	–	–	–	40	19	2	11
	Non-PROMs	–	–	–	–	76	36	1	5

*PCT* palliative care team; *PCU* palliative care unit; *PRO* patient-reported outcome; *PROMs* patient-reported outcome measures

Table 2 shows the PROMs in use at institutions that reported routine use of PROMs in palliative care clinical practice. The most commonly used PROM instrument was the CCNSq, which was used by the PCT at 20 institutions (57%) and at outpatient palliative care services at 54 institutions (82%). For PCUs, the CCNSq was routinely used at 10 institutions (42%), whereas the Distress and Impact Thermometer was used at 12 institutions (50%). The CCNSq includes the Distress and Impact Thermometer, and some institutions had multiple responses. The QOL scale was used by one home hospice only (5%).

**Table 2:** PROMs routinely used in palliative care clinical practice

	Designated cancer hospital (n = 83)				PCUs (n = 24)		Home hospices (n = 1)	
	PCTs (n = 35)		Outpatient palliative care (n = 66)					
	n	%	n	%	n	%	N	%
CCNSq	20	57	54	82	10	42	0	0
Thermometer	13	37	31	47	12	50	0	0
ESAS	7	20	7	11	4	17	0	0
IPOS	3	9	3	5	0	0	0	0
QOL scale	2	6	0	0	2	8	1	100
MDASI	0	0	2	3	1	4	0	0
PRO-CTCAE	1	3	1	2	–	–	–	–

*CCNSq* Comprehensive Care Needs Survey questionnaire; *ESAS* Edmonton Symptom Assessment System; *IPOS* Integrated Palliative care Outcome Scale; *MDASI* MD Anderson Symptom Inventory; *PCT* palliative care team; *PCU* palliative care unit; *PROMs* patient-reported outcome measures; *PRO-CTCAE* patient-reported outcomes version of the common terminology criteria for adverse events; *QOL* quality of life

#### Attitude towards PROs

Table 3 shows the opinions about PROs of healthcare providers at institutions that routinely using PROMs. Of the 108 institutions routinely using PROMs, 99 respondents (92%) stated that these instruments were useful in symptom management (representing a higher prevalence than the perceptions at the institutions that did not routinely use PROMs; p = 0.002). Further, 37 of the respondents (34%) that routinely used PROMs and 143 of the respondents (41%) that did not routinely use PROMs had negative opinions in regard to the burden placed on patients through the use of these instruments. There was also a significant difference in opinions regarding burdens on healthcare providers between respondents that did or did not routinely use PROMs (p = 0.007). Moreover, > 50% of the respondents that routinely used PROMs stated (via the surveyed healthcare providers employed therein) that patients cannot be assessed when their disease progresses and that results are influenced by the patient’s level of cognitive function. Fewer than 10% of healthcare providers responded that the healthcare provider can evaluate outcomes even if the patient does not self-evaluate (i.e., reflecting a paternalistic view of medical care).

**Table 3:** Opinions about PROs of healthcare providers in institutions that routinely use PROMs

	Participants routinely using PROMs (n = 108)		Participants not routinely using PROMs (n = 350)		p-value
	n	%	n	%	
Useful in symptom management	99	92	276	79	0.002*
Patients cannot be assessed when their disease progresses	74	69	260	74	0.27
Influenced by the patient's level of cognitive function	57	53	215	61	0.12
Burdensome on patients	37	34	143	41	0.26
Time consuming in terms of explaining how to complete the form	25	23	77	22	0.79
Burdensome on healthcare providers	27	25	47	13	0.007*
Worries about addressing the reported symptoms	20	19	41	12	0.08
Healthcare provider can evaluate outcomes even if the patient does not self-evaluate	6	6	30	9	0.41
Not useful for symptom management	2	2	14	4	0.38

*PROs* patient-reported outcomes, *PROMs* patient-reported outcome measures

*Respondents had the option to choose multiple selections

### Interview

#### Institution characteristics

A total of 24 institutions that responded to the questionnaire agreed to be interviewed: 13 designated cancer hospitals (54%), nine PCUs (38%), and two home hospices (8%). The respondents held a range of positions: 11 were physicians (42%), 13 were nurses (50%), one was a pharmacist (4%), and one was a psychologist (4%). Of the respondents 15 (63%) routinely used PROMs, whereas three had discontinued their use.

#### Assessment of the usefulness, barriers, and attitudes of healthcare provider towards PROMs

Table 4 shows respondents’ views on the usefulness of PROMs, the barriers to their use, and the practical effective methods used for PROMs. Responses were recorded for usefulness and barriers for patients and healthcare providers regarding the perspective of healthcare providers. Patients reported usefulness in regard to facilitating communication with healthcare providers (n = 8) and improving self-care skills (n = 13), while healthcare providers’ determination of usefulness was in regard to gaining a better understanding of the patient's condition and using the findings of PROMs in the provision of more optimal care (n = 25), building good relationships with patients (n = 6), and educational benefits to healthcare providers (n = 7).

**Table 4:** Views on the usefulness of and barriers to using PROMs and relevant effective methods

Views	Theme (n)			n (%)
Usefulness of PROMs	Patients	Improving self-care skills	Gaining an understanding of their own symptoms and changes in symptoms (n = 7)	13 (22%)
			Participating in treatment and care with a sense of self-management (n = 4)	
			Having a sense of self-control (n = 2)	
		Facilitating communication with healthcare providers	Opportunity to talk about one's thoughts and feelings to healthcare providers (n = 6)	8 (13%)
			Feeling that one's healthcare provider is interested in you (n = 2)	
	Healthcare providers	Gaining an understanding of patients' conditions	Understanding the patient's pain and condition (n = 17)	25 (42%)
			Using this information in patient treatment and care (n = 5)	
			Sharing information among healthcare providers (n = 3)	
		Building good relationships with patients	Facilitating communication with patients (n = 5)	6 (10%)
			Building good relationship with patients (n = 1)	
		Educational benefits for healthcare providers	Training in assessing the scale (n = 6)	7 (12%)
			Facilitating grief work (n = 1)	
		Use in research	Useful for research applications (n = 1)	1 (2%)
Barriers related to the use of PROMs	Patients	Difficulty using PROMs due to incomprehension or cognitive decline	Need a better understanding and more communication about what patients are being asked to assess (n = 13)	34 (33%)
			Difficulty in numerical evaluation (n = 12)	
			Difficulty with navigating PROs due to old age or a decline in cognitive function (n = 9)	
		Difficulty using PROMs due to worsening symptoms or a decreased state of consciousness	Difficulty in assessing results due to symptoms of disease progression (n = 5)	8 (8%)
			Difficulty with PROs due to delirium or a decreased state of consciousness (n = 3)	
		Burdensome on patients	Burden due to use of PROMs (n = 4)	5 (5%)
			Burden from repeated assessments (n = 1)	
		Difficulty filling out PROMs due to disease progression	Difficulty in filling out the form due to symptoms or state of consciousness (n = 3)	4 (4%)
			Distress due to not being able to fill out the form by oneself (n = 1)	
	Healthcare providers	Burden on healthcare providers	Burden due to increased workload (n = 6)	20 (20%)
			Difficulty in quantifying PROs (n = 6)	
			Difficulty in ensuring sufficient time for PROs (n = 5)	
			Conflict in regard to quantifying assessments as patients' conditions deteriorate (n = 3)	
		Lack of staff education on the use of PROMs	Insufficient education of healthcare providers on the use of PROMs (n = 7)	12 (12%)
			Insufficient education of healthcare providers with respect to PROs (n = 5)	
		Difficulty in using PROMs and in linking these measures to care decisions	Difficulties in linking PRO results to care decisions (n = 12)	15 (15%)
			Difficulties in regard to the timing of PROs (n = 3)	
		Need for guidance in the evaluation of PROMs	Lack of comprehensive assessment available from the use of PROMs (n = 3)	4 (4%)
			Difficulties evaluating the results obtained from the implementation of PROMs (n = 1)	
Practical effective methods relevant to the use of PROMs	Efforts to reduce the burden on patients	Devising evaluation methods tailored to the patient's condition (n = 20)	45 (41%)	
		Selecting PROMs according to the patient's condition (n = 14)		
		Consideration of the patients most suitable for the use of PROMs (n = 11)		
	Education on using PROMs for healthcare providers	Education on how to use PROMs in clinical practice (n = 14)	21 (19%)	
		Education how to use PROMs in general (n = 7)		
	Standard procedures for using PROMs	Rules for using PROMs (n = 9)	28 (25%)	
		Rules for utilizing the results of PROM evaluations (n = 7)		
		Preparing for the introduction of PROMs (n = 6)		
		Education on how to describe the results of PROM evaluations in the clinical record (n = 6)		
	Awareness and consideration of healthcare providers involved in the evaluation of PROs	Considerations when using PROMs (n = 5)	16 (15%)	
		Awareness of staff involved in the use of PROs (n = 11)		

*PROs* patient-reported outcomes; *PROMs* patient-reported outcome measures

In regard to barriers to the use of PROMs, the most common patient-reported barriers included difficulty in using PROMs due to incomprehension or cognitive decline (n = 34), difficulty using PROMs due to worsening symptomology or a decreased state of consciousness (n = 8), the burden placed on patients (n = 5), and difficulty in filling out the form by themselves due to progression of their disease (n = 4). The barriers reported by healthcare providers included the burden placed on healthcare providers (n = 20), difficulty in using PROMs and linking the findings of these reports to care decisions (n = 15), the need for more comprehensive healthcare provider education on the use of PROMs (n = 12), and the need for training in regard to the evaluation and interpretation of PROMs (n = 4).

We also asked about practical effective methods employed in the use of PROMs. Respondents reported that practical effective methods had been implemented in efforts to reduce the burden placed on patients (n = 45), improving healthcare providers’ education on the use of PROMs (n = 21), implementing standard procedures for the use of PROMs (n = 28), and increasing awareness and consideration towards healthcare providers involved in the evaluation of PROs (n = 16).

## Discussion

This study is the first report of the routine use of PROs in SPC in Japan. This study showed that (1) only 108 institutions (24%) routinely used PROMs in SPC in Japan, (2) the most frequently used PROM instrument was the CCNSq, and (3) most institutions using PROMs responded that they were useful (i.e., via representative healthcare providers).

In SPC, PROMs were used somewhat more frequently in palliative care outpatient clinics (29%) than in other settings. However, PROMs were used less routinely in PCUs and were rarely used in home hospice care (5%). In palliative care clinical practice, it is estimated that approximately 60% of patients are unable to complete PROMs [11]. This is because patients receiving palliative care have deteriorating medical conditions and experience cognitive decline; this challenge was also mentioned as a barrier to use of PROMs in the current study. Previous study have reported a decrease in the percentage of patients using PROMs as their conditions became more severe [20]. Moreover, patients who are able to go to outpatient clinics are more likely to use PROMs than those who are hospitalized simply because they by definition have a greater state of health and ability (i.e., they are able to attend outpatient clinics), and PROMs can effectively be used to assess and alleviate their symptomology [20].

As evidenced in the current evaluation, the most frequently used PROM was the CCNSq. The CCNSq is a tool developed in Japan specifically in order to screen for distress in cancer patients for the purpose of deciding on the early introduction of palliative care; this instrument has been shown to be both reliable and valid [15, 16]. Although the CCNSq does not include questions on spiritual aspects, it is considered to be a widely used and popular option because it allows for the assessment of a patient's condition from multiple perspectives. The CCNSq can be used as a screening instrument for patient distress independent from decisions regarding early introduction of palliative care.

Many PROMs have been developed worldwide. One instrument, the ESAS, has been translated into more than 20 languages; however, the items mainly concern physical symptomology, and it is therefore difficult to conduct a comprehensive assessment using this tool [21]. However, the choice of PROMs varies from institution to institution and reasons for selection vary by institution. It is necessary and critically important to use a tool that is not burdensome to the patient and can comprehensively assess distress.

In the present study, 92% of institutions that routinely used PROMs reported that these instruments are useful in symptom management, a significant difference from the positive response rate among institutions that did not routinely use PROMs. Moreover, according to the study interview, the reason given for the usefulness of PROMs was that this methodology facilitated communication between patients and healthcare providers. We believed that the use of PROMs allows patients to talk to healthcare providers about their symptoms and concerns, and facilitates communication [22]. As a result, it likewise helped healthcare providers to better understand patients’ symptoms and conditions. We consider that gaining a better understanding of patients’ conditions may lead to a better assessment of care goals (as reported in previous studies) [23]. From these results, we can infer that the use of PROMs allows providers to appropriately assess patients' conditions as well as to provide proper care management for their symptomology. However, institutions that routinely use PROMs did not only respond affirmatively in regard to their usefulness, but also reported in regard to the burden placed on patients and healthcare providers as a result of their implementation. In this study, we did not ask patients directly, so the actual burden of using PROMs for patients is not clear. Patients are the experts on their experience with their diseases, and we believe that patient self-reporting can provide unbiased information and help identify symptom and needs and improve care. In addition, a study of healthcare providers at a cancer center reported that the strengths outweigh the weaknesses, such as the possibility for patients to express their health condition and the facilitation of communication between patients and healthcare providers through the use of PROMs [24]. We consider that the use of PROMs and/or Patient-Reported Experience measures (PREMs) may be useful to patients and that we need to examine ways to reduce the burden use of these tools [25].

More specifically, the results from the interview administered in the present study revealed barriers in the use of PROMs not only for patients but also for healthcare providers. We consider that this needs to be overcome, as evidence by the barriers to PRO implementation, such as concerns about the increase work in using PROMs and lack of training on the use of the tool.

As reported in previous studies, we conclude that the use of PROMs for patients admitted to PCUs requires that providers understand that only some patients should be targeted for provision of this self-report methodology [26]. In patients who are eligible in palliative care setting, the situation may make it difficult to response PROMs due to their condition. In this survey, more than 50% of the respondents answered that PROs were difficult to use due to the progression of the patient’s disease or cognitive decline. On the other hand, Seipp et al. reported the use of PROs may be feasible if the patient’s condition is carefully considered with the use of PROMs [27]. The careful selection of eligible patients may reduce barriers to the use of PROMs that occur as a result of patient conditions (i.e., patients may be screened as to their state of health and the likely burden that the use of PROMs would entail). We strongly recommend that these modifications and nuances be communicated in the education and training of healthcare providers. It is important that healthcare providers are trained in the use of PROs and how they can be used in their care, rather than thinking that it is impossible for all patients to use PROs because they are vulnerable, such as patient in PCUs to reduce barriers. This survey did not directly ask patients, so the actual burden on patients from use of PRMs is not clear. However, we consider that PROMs can be used even if the burden is caused, through patients and healthcare providers recognition that PROs are provided to patients for their care.

We note that, although the present survey did not directly ask patients about patient burden as a barrier in the use of PROs, healthcare providers responded affirmatively in this regard. Hence, the current lack of validation regarding the effectiveness of PROs in SPC may lead healthcare providers to erroneously conclude that PROs are a burden to patients. Therefore, studies providing a high level of evidence in support of the effectiveness of PROMs (or the lack thereof), including gold-standard RCTs, are needed in the future.

In previous studies, healthcare providers were concerned about the burden that PROs represented to themselves in regard to the need for increased explanation and assistance in filling out PROMs when administering these tools in clinical practice, and about how to respond to the identification of symptoms and needs derived from these PROMs; these previously reported concerns [28–30] are consistent with the results of the present study. Therefore, it is necessary to create a system that enables healthcare providers to provide treatment and care for patients' identified symptoms and needs in order to reduce the burden placed on healthcare providers.

In this study, the development of standard procedures for the use of PROMs and increased education and training for healthcare providers were suggested as ways to utilize PROs more effectively in palliative care practice. It is necessary to not only reduce the burden on patients in the utilization of PROMs, but to also address burden reduction (including in terms of relevant education and training) for healthcare providers [26, 31, 32]. Currently, an implementation method for PROs that considers the burden placed on patients and on healthcare providers has not yet been established. Therefore, future research is needed in order to establish an effective implementation method. Moreover, in the future, it will be important to accumulate and share know-how and expertise, such as in regard to the results of surveys on how PROMs are currently being implemented in palliative care clinics and the effective methods that institutions have successfully implemented in administering PROMs.

We note that, in the present study, there were relatively few opinions reported among the survey results that reflected the more traditional paternalistic view that healthcare providers can evaluate symptoms and outcomes even if the patient cannot self-evaluate. In some circumstances, a proxy evaluation by a healthcare provider may be useful, but symptoms and psychosocial aspects that are invisible to others may cause discrepancies in evaluation between the patient and the healthcare provider. In fact, it is clear that PROMs are not widely used in palliative care clinical practice, and therefore such responses may have been underreported due to social desirability bias. More specifically, it is likely that non-PROM instruments have been routinely used instead of PROMs in palliative care practice due to Japanese cultural characteristics that favor a paternalistic approach [11, 33]. However, since there has been a shift from a paternalistic approach to patient-participatory medicine within Japanese healthcare during these past decades [34], it is possible that social desirability bias may have influenced the study results to a small extent [35]. In Japan, it was found that PROs are not routinely used in clinical palliative care compared to other countries, such as Australia, where PROs are focused and implemented in clinical palliative care [36]. We believed it is necessary to implement PROs in the future to improve the quality of palliative care. This study revealed the use of PROMs and barriers in SPC, but we believe that further RCTs on symptom management, facilitation of patient-healthcare provider communication, and overall survival need to be conducted in order to clarify the effectiveness of PROMs.

### Study limitations

One limitation of this study is that the collection rate for the administered questionnaire was less than 50%. Therefore, our results may not reflect the actual situation in regard to the use of PROMs in palliative Japan as a whole, and the overall generalizability of our findings is unclear. Moreover, we evaluated only 19 home hospices in the questionnaire survey, which might not be sufficient to reflect the situation in those settings or in the professional palliative care field in general. The second limitation is that we did not research palliative care for people with other diseases. Therefore, the results do not show the overall results of palliative care.

## Conclusions

This study is the first to report the routine use of PROs in SPC in Japan. In sum, the survey administered in the present study quantified the status of the routine use of PROMs in SPC in Japan, revealed barriers to the wider use of PROMs in this country, and identified needed innovations in the implementation of this assessment methodology. Based on the results of the present study, it is necessary to consider the usefulness of PROs in clinical palliative care, as well as the careful selection of PROMs according to the patient's condition and how specifically to introduce and operate PROMs in order to minimize the resulting burden on patients and healthcare providers. In addition to cross-sectional questionnaires and interviews evaluating the perceptions of both patients and healthcare providers, studies objectively evaluating the usefulness of PROMs (including in RCTs) are needed in the future.

## Supplementary Information

**Additional file 1**. **Table S1**: Items of the questionnaire, and **Table S2**: Interview guide.

## Data Availability

Data supporting the results of this study are not available to the public. However, they are available from the Division of Palliative Care Nursing at the Department of Health Sciences (Tohoku University Graduate School of Medicine) upon reasonable request to the corresponding author or the division administrators (http://www.pn.med.tohoku.ac.jp).

## Abbreviations

CCNSq: Comprehensive Care Needs Survey questionnaire
DIT: Distress and Impact Thermometer
EORTC QLQ C-30: European Organization for Research and Treatment of Cancer Quality of Life Questionnaire-Core 30
ESAS: Edmonton Symptom Assessment System
IC: Integrated circuit
IPOS: Integrated Palliative care Outcome Scale
MDASI: MD Anderson Symptom Inventory
NRS: Numerical Rating Scale
PCT: Palliative care team
PCU: Palliative care unit
POS: Palliative care outcome score
PRO: Patient-reported outcome
PRO-CTCAE: PRO version of the common terminology criteria for adverse events
PROMs: Patient-reported outcome measures
QOL: Quality of life
RCT: Randomized controlled trial
SPC: Specialized palliative care
STAS: Support Team Assessment Schedule

## References

[1] U.S. Food and Drug Administration (2009) Patient-reported outcome measures: use in medical product development to support labeling claims. https://www.fda.gov/media/77832. Accessed 30 Mar 2022.

[2] Basch E (2010). The missing voice of patients in drug-safety reporting. N Engl J Med.

[3] Laugsand EA, Sprangers MAG, Bjordal K (2010). Health care providers underestimate symptom intensities of cancer patients: a multicenter European study. Health Qual Life Outcomes.

[4] Chen J, Ou L, Hollis SJ (2013). A systematic review of the impact of routine collection of patient-reported outcome measures on patients, healthcare providers and health in an oncologic setting. BMC Health Serv Res.

[5] Snyder CF, Aaronson NK, Choucair AK (2012). Implementing patient-reported outcome assessment in clinical practice: a review of the options and considerations. Qual Life Res.

[6] Fung CH, Hays RD (2008). Prospects and challenges in using patient-reported outcome in clinical practice. Qual Life Res.

[7] Basch E, Deal AM, Kris MG (2016). Symptom monitoring with patient-reported outcomes during routine cancer treatment: a randomized controlled trial. J Clin Oncol.

[8] Etkind SN, Daveson BA, Kwok W (2015). Capture, transfer, and feedback of patient-centered outcomes data in palliative care populations: does it make a difference? A systematic review. J Pain Symptom Manag.

[9] Graupner C, Kimman ML, Mul S (2021). Patient outcomes, patient experiences and process indicators associated with the routine use of patient-reported outcome measures (PROMs) in cancer care: a systematic review. Support Care Cancer.

[10] Yount SE, Rothrock N, Bass M (2014). A randomized trial of weekly symptom telemonitoring in advanced lung cancer. J Pain Symptom Manag.

[11] Dudgeon D (2018). The impact of measuring patient-reported outcome measures on quality of and access to palliative care. J Palliat Med.

[12] Carduff E, Johnston S, Winstanley C (2018). What does ‘complex’ mean in palliative care? Triangulating qualitative findings from 3 settings. BMC Palliat Care.

[13] Collins ES, Witt J, Bausewein C, Daveson BA (2015). A systematic review of the use of the Palliative Care Outcome Scale and the support team assessment schedule in palliative care. J Pain Symptom Manag.

[14] Okuyama T, Kizawa Y, Morita T (2016). Current status of distress screening in designated cancer hospitals: a cross-sectional nationwide survey in Japan. J Natl Compr Cancer Netw.

[15] Okuyama T, Wang XS, Akechi T (2003). Japanese version of the MD Anderson symptom inventory: a validation study. J Pain Symptom Manag.

[16] Akizuki N, Akechi T, Nakanishi T (2003). Development of a brief screening interview for adjustment disorders and major depression in patients with cancer. Cancer.

[17] Sakurai H, Miyashita M, Imai K (2019). Validation of the Integrated Palliative care Outcome Scale (IPOS) – Japanese version. Jpn J Clin Oncol.

[18] Tsuneto S (2013). Past, present, and future of palliative care in Japan. Jpn J Clin Oncol.

[19] Nowell L, Norris JM, White DE (2017). Thematic analysis: striving to meet the trustworthiness criteria. Int J Qual Methods.

[20] Kang JH, Kwon JH, Hui D (2013). Changes in symptom intensity among cancer patients receiving outpatient palliative care. J Pain Symptom Manag.

[21] Bruera E, Kuehn N, Miller MJ (1991). The Edmonton Symptom Assessment System (ESAS): a simple method for the assessment of palliative care patients. J Palliat Care.

[22] Greenhalgh J, Gooding K, Gibbons E (2018). How do patient reported outcome measures (PROMs) support clinician-patient communication and patient care? A realist synthesis. J Patient Rep Outcomes.

[23] Goldberg SL, Paramanathan D, Khoury R (2019). A patient-reported outcome instrument to assess symptom burden and predict survival in patients with advanced cancer: flipping the paradigm to improve timing of palliative and end-of-life discussions and reduce unwanted health care costs. Oncologist.

[24] Brunelli C, Zito E, Alfieri S (2022). Knowledge, use and attitudes of healthcare professionals towards patient-reported outcome measures (PROMs) at a comprehensive cancer center. BMC Cancer.

[25] Weldring T, Smith SM (2013). Patient-reported outcomes (PROs) and patient-reported outcome measures (PROMs). Health Serv Insights.

[26] Coym A, Ullrich A, Hackspiel LK (2020). Systematic symptom and problem assessment at admission to the palliative care ward – perspectives and prognostic impacts. BMC Palliat Care.

[27] Seipp H, Haasenritter J, Hach M (2022). Integrating patient- and caregiver-reported outcome measures into the daily care routines of pecialized outpatient palliative care: a qualitative study (ELSAH) on feasibility, acceptability and appropriateness. BMC Palliat Care.

[28] Velikova G, Booth L, Smith AB (2004). Measuring quality of life in routine oncology practice improves communication and patient well-being: a randomized controlled trial. J Clin Oncol.

[29] Bausewein C, Daveson BA, Currow DC (2016). EAPC white paper on outcome measurement in palliative care: improving practice, attaining outcomes and delivering quality services—recommendations from the European Association for Palliative Care (EAPC) task force on outcome measurement. Palliat Med.

[30] Yang LY, Manhas DS, Howard AF (2018). Patient-reported outcome use in oncology: a systematic review of the impact on patient–clinician communication. Support Care Cancer.

[31] Antunes B, Harding R, Higginson IJ (2014). Implementing patient-reported outcome measures in palliative care clinical practice: a systematic review of facilitators and barriers. Palliat Med.

[32] Murtagh FE, Ramsenthaler C, Firth A (2019). A brief, patient- and proxy-reported outcome measure in advanced illness: validity, reliability and responsiveness of the Integrated Palliative care Outcome Scale (IPOS). Palliat Med.

[33] Claramita M, Nugraheni MDF, van Dalen J (2013). Doctor–patient communication in Southeast Asia: a different culture?. Adv Health Sci Educ.

[34] Slingsby BT (2004). Decision-making models in Japanese psychiatry: transitions from passive to active patterns. Soc Sci Med.

[35] Krumpa I (2013). Determinants of social desirability bias in sensitive surveys: a literature review. Qual Quant.

[36] Palliative Care Outcomes Collaboration. https://www.uow.edu.au/ahsri/pcoc/. Accessed 30 Dec 2022.

