# Supplementary material for: Routine patient assessment and the use of patient-reported outcomes in specialized palliative care in Japan
Source: J Patient Rep Outcomes. 2023 Mar 9;7:25. doi: 10.1186/s41687-023-00565-z (PMC9998740; doi:10.1186/s41687-023-00565-z)
Supplement: Supplementary file 1 — Additional file 1. Table S1: Items of the questionnaire, and Table S2: Interview guide. [file 41687_2023_565_MOESM1_ESM.docx]

| **APPENDIX** |  |  |  |  |  |  |
| --- | --- | --- | --- | --- | --- | --- |

Appendix Table1. Items of questionnaire

| PROMs/Non-PROMs routinely used in palliative care clinical practice: | | |  |
| --- | --- | --- | --- |
| Please select all choices that your institution routinely use to assess symptoms and other conditions of patients receiving palliative care | | | |
|  | Designated cancer hospitals | PCUs | Home hospices |
| Non-PROMs | -Palliative care outpatient | -Admission to PCU | -The first medical examination |
| STAS-J | -Routine PCT assessment | -Routine assessment | -Routine assessment |
| IPOS | -Admission distress screening | -Palliative care outpatient | -Calculation of cancer patient  management fee |
| PROMs | -Continuing distress screening | -Other |  |
| Pain scale (NRS, VAS, FS) | -Calculation of cancer patient  management fee |  |  |
| IPOS |  |  |  |
| CCNSq | -Other |  |  |
| ESAS |  |  |  |
| MDASI |  |  |  |
| QOL scale |  |  |  |
| Thermometer |  |  |  |
| PRO-CTCAE |  |  |  |
| Others |  |  |  |

| Thoughts on PROs: |  |
| --- | --- |
| Please select all choices that apply to you about using of PROs | Useful in symptom management |
|  | Not useful for symptom management |
|  | Burdensome on patients |
|  | Burdensome on healthcare providers |
|  | Patients cannot be assessed when their disease progresses |
|  | Influenced by the patient's level of cognitive function |
|  | Time consuming in terms of explaining how to complete the form |
|  | Healthcare provider can evaluate outcomes even if the patient does not self-evaluate |
|  | Worries about addressing the reported symptoms |
|  | Others |
| Experience with PROMs in the past: |  |
| 1) Please select a choice that apply to your institution | PROMs was previously used but discontinued |
|  | Never used / continuing to use PROMs |
| 2) Please select all choices that reasons for discontinuing use of PROMs (Only institution chose to discontinued to use PROMs) | Could not accurately assess |
|  | Patients did not want to complete PROMs |
|  | Patients could not be assessed when their disease progresses |
|  | Burdensome on patients |
|  | Did not know about the appropriate PROMs |
|  | Completing PROMs became an objective |
|  | It took time to explain how to fill out PROMs |
|  | Healthcare providers requested that PROMs be discontinued |
|  | There was no consensus within the institution |
|  | Evaluation could be made without using PROMs |
|  | Changed to another scale |
|  | Others |
| 3) Please select a choice that apply to your institution | Non-PROMs such as STAS-J was previously used but discontinued |
|  | Never used / continuing to use Non-PROMs such as STAS-J |
| 4) Please select a choice that apply to your institution (Only institution chose to discontinued to use Non-PROMs) | Could not accurately assess |
|  | Healthcare provider's values affected the score |
|  | It took time to assess Non-PROMs |
|  | Did not have the opportunity to share with other professionals in the institution |
|  | Healthcare providers requested that Non-PROMs be discontinued |
|  | Did not know how to use PROMs smoothly and effectively |
|  | There was no consensus within the institution |
|  | Completing PROMs became an objective |
|  | Changed to another scale |
|  | Others |

Appendix Table2. Interview guide

| Interview guide |
| --- |
| ・Please tell me about your experience using PROs that you have thought were useful? |
| ・What barriers or challenges do you think PROs face in your palliative care clinical practice? |
| ・What are your efforts to implement PROMs in your palliative care clinical practice? |
